# Biomedical Big Data Technologies, Applications, and Challenges for Precision Medicine: A Review

**DOI:** 10.1002/gch2.202300163

**Published:** 2023-11-20

**Authors:** Xue Yang, Kexin Huang, Dewei Yang, Weiling Zhao, Xiaobo Zhou

**Affiliations:** ^1^ Department of Pancreatic Surgery and West China Biomedical Big Data Center West China Hospital Sichuan University Chengdu 610041 China; ^2^ College of Advanced Manufacturing Engineering Chongqing University of Posts and Telecommunications Chongqing Chongqing 400000 China; ^3^ Center for Systems Medicine School of Biomedical Informatics UTHealth at Houston Houston TX 77030 USA

**Keywords:** biomedical big data, electronic medical record, federated learning, knowledge graph, medical imaging analysis, omics data, precision medicine

## Abstract

The explosive growth of biomedical Big Data presents both significant opportunities and challenges in the realm of knowledge discovery and translational applications within precision medicine. Efficient management, analysis, and interpretation of big data can pave the way for groundbreaking advancements in precision medicine. However, the unprecedented strides in the automated collection of large‐scale molecular and clinical data have also introduced formidable challenges in terms of data analysis and interpretation, necessitating the development of novel computational approaches. Some potential challenges include the curse of dimensionality, data heterogeneity, missing data, class imbalance, and scalability issues. This overview article focuses on the recent progress and breakthroughs in the application of big data within precision medicine. Key aspects are summarized, including content, data sources, technologies, tools, challenges, and existing gaps. Nine fields—Datawarehouse and data management, electronic medical record, biomedical imaging informatics, Artificial intelligence‐aided surgical design and surgery optimization, omics data, health monitoring data, knowledge graph, public health informatics, and security and privacy—are discussed.

## Graphical abstract

The explosive growth of biomedical Big Data presents both significant opportunities and challenges in the realm of knowledge discovery and translational applications within precision medicine. Efficient management, analysis, and interpretation of big data can pave the way for groundbreaking advancements in precision medicine. The review focuses on the recent progress and breakthroughs in the application of big data within precision medicine.

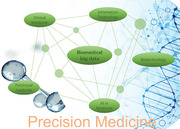

## 1 Introduction

The explosive expansion of biomedical big data has ushered in a wealth of opportunities and challenges in the domains of knowledge discovery within biomedical big data and its translational applications in precision medicine. Within the realm of precision medicine, biomedical big data is considered one of the most crucial and challenging components. Precision medicine aims to provide tailored medical solutions for each patient based on factors such as their genetic information, physiological characteristics, and lifestyle. To achieve this goal, a comprehensive understanding of individuals is required, and biomedical big data serves as the foundation for gaining insights into the intricacies of each person's life. First, biomedical big data includes genomics data, proteomics data, and metabolomics data, which reveal individuals' genetic and molecular traits. By analyzing these data sets, doctors and researchers can identify genetic susceptibilities, drug response patterns, and potential genetic mutations, laying the groundwork for personalized treatment plans. Additionally, biomedical big data includes clinical data, electronic medical records (EMRs), and medical imaging data, providing detailed information about patients' medical histories and physical conditions. These data can be used for disease prediction, early diagnosis, and treatment monitoring. Furthermore, biomedical big data incorporates health monitoring data supported by sensors and Internet of Things (IoT) technologies. These data can continuously monitor a patient's physiological status, offering real‐time health information that aids in disease prevention and chronic disease management.

Therefore, biomedical big data is of paramount importance in realizing personalized medicine, disease prediction, treatment optimization, and medical decision support. It provides critical information for research in precision medicine, enabling us to better understand individual health conditions and solidify the foundation for personalized healthcare and health management.

In this review, we mainly discuss the following topics related to big data science for precision medicine, as well as the challenges faced in processing and handling biomedical big data.

1. Datawarehouse and data management.
2. EMR for precision medicine.
3. Biomedical imaging informatics for translational research and clinical diagnosis and treatment.
4. AI‐aided surgical design and surgery optimization.
5. Applications of omics data for precision medicine in bioinformatics.
6. Health monitoring data for precision medicine.
7. Knowledge graph for precision medicine.
8. Public health informatics.
9. Security and privacy of precision medicine data.

## 2 Datawarehouse and Data Management

Precision medicine is an emerging field that aims to tailor medical treatments to individual patients based on their unique genetic makeup, environment, and lifestyle. To effectively manage the large amount of data generated by precision medicine, database technology, and data management methods play a critical role.

One of the key requirements for precision medicine is the ability to manage and integrate large amounts of diverse data types from different sources, including genomics, imaging, clinical, and environmental data. This requires the use of specialized database technologies that can handle complex data structures, support flexible querying, and enable efficient data integration and sharing.

1. Big Data Platforms: big data platforms such as Apache Hadoop^[^ ^1^ ^]^ and Apache Spark^[^ ^2^ ^]^ are used to store and process this data. These platforms enable distributed storage and processing of large‐scale genomic,^[^ ^3^, ^4^, ^5^ ^]^ clinical, and imaging data,^[^ ^6^, ^7^, ^8^ ^]^ making it easier to extract meaningful insights.
2. Cloud Computing: Cloud computing is becoming increasingly popular in precision medicine as it enables researchers and clinicians to store and analyze large amounts of data in the cloud without the need for on‐premises infrastructure.^[^ ^9^, ^10^ ^]^ Cloud providers such as Amazon Web Services (AWS),^[^ ^11^ ^]^ Microsoft Azure,^[^ ^12^ ^]^ and Google Cloud Platform offer a range of services and tools that can be used to store, process, and analyze precision medicine data.^[^ ^10^, ^13^ ^]^
3. NoSQL Databases^[^ ^14^ ^]^: NoSQL databases such as MongoDB,^[^ ^15^, ^16^, ^17^ ^]^ Cassandra,^[^ ^15^, ^18^ ^]^ and HBase^[^ ^19^ ^]^ are used to store and manage large‐scale genomic and clinical datasets. These databases offer scalability, flexibility, and high availability, making them well‐suited to handle the complex and dynamic nature of precision medicine data. They use graph data models to represent data as nodes, edges, and properties. This enables the representation of complex relationships between different data entities, such as genes, proteins, diseases, and drugs, and supports powerful querying capabilities, such as pattern matching and traversal. Graph databases are well‐suited for precision medicine applications because they can handle large and heterogeneous datasets and provide a flexible and scalable platform for data integration and analysis.
4. Data Integration and Interoperability Platforms: Clinicians and data scientists often ask where and how to extract the data from a hospital's database when deciding to study a specific disease. In the healthcare industry, the sources of big medical data include hospital and pharmaceutical enterprise records, medical records of patients, results of medical examinations, medical costs, social security records, etc. Data integration and interoperability platforms such as OMOP^[^ ^20^, ^21^ ^]^ and SMART Health IT^[^ ^22^, ^23^ ^]^ are used to integrate and harmonize data from multiple sources, enabling researchers and clinicians to access and analyze data seamlessly.

The core of data flow in the data management system is the Translational Data Warehouse (TDW)^[^ ^24^ ^]^ based on Informatics for Integrating Biology & the Bedside (I2B2) core technology.^[^ ^25^ ^]^ TDW aggregates data from pre‐clinical and clinical data sources. Pre‐clinical data resources include primate centers, pre‐clinical imaging, and shared resource cores.^[^ ^26^ ^]^ Clinical and research data sources include clinical electronic medical records such as Epic,^[^ ^27^ ^]^ and Public Health Sciences’ research databases, clinical research imaging, clinical registries, and data from various clinic centers.^[^ ^24^ ^]^ The I2B2 framework^[^ ^25^ ^]^ is a platform designed to support the integration and analysis of heterogeneous biomedical data, and it uses ETL processes to extract data from various sources, transform it into a standard format, and load it into the data warehouse. ETL processes are used to integrate data from different sources, such as electronic health records, imaging systems, genomic databases, and so on. Besides, the i2b2 framework provides a diverse set of analytical tools enabling researchers and clinicians to delve into and analyze the data stored in the data warehouse. These tools include visualizations, statistical analysis tools, and machine learning algorithms.^[^ ^25^ ^]^

Hospital databases like the EPIC system have a certain search function, but it is primarily designed for general searches and may not suffice for complex criteria. Therefore, we often need to devise algorithms tailored to clinicians' specific requirements. This customization can be achieved within the clinical database framework. The I2B2 team has developed another search and visualization tool called “shrine”^[^ ^28^ ^]^ that builds upon the I2B2 core functionality. By utilizing this tool to identify suitable patients based on specific criteria, doctors can further validate these patients by reviewing their records. This process aids in determining whether a patient is a suitable candidate for the next steps of treatment or research. It is worth pointing out that other tools like Redcap,^[^ ^29^, ^30^, ^31^ ^]^ Hospital Information System (HIS), Observational Health Data Sciences and Informatics (OHDSI),^[^ ^32^ ^]^ and others are also very good.

Another important aspect of data management for precision medicine is the adoption of data standards and ontologies. These are standardized representations of data concepts and relationships that enable data interoperability and facilitate data sharing and reuse across different domains and applications. For example, the Clinical Data Interchange Standards Consortium (CDISC) provides standard formats for clinical trial data,^[^ ^30^, ^33^ ^]^ and the Human Phenotype Ontology (HPO)^[^ ^34^ ^]^ provides a standardized vocabulary for describing human phenotypes. The Clinical Data Interchange Standards Consortium (CDISC) is a non‐profit organization that develops and promotes global standards for clinical research data. CDISC standards provide a common language for describing and sharing clinical data, enabling data integration and analysis across multiple studies and domains.^[^ ^33^ ^]^

Additionally, data governance is also critical for precision medicine. This involves the development of policies and procedures for data access, security, privacy, and ethical use. As precision medicine involves the use of sensitive patient data, it is important to ensure that data is collected, stored, and used in a secure and ethical manner.

The biomedical research community generates large volumes of digital data in different formats using different modalities. These data should be findable, accessible, interoperable, and reusable (FAIR).^[^ ^35^ ^]^ Thus, scientists can take full advantage of them. The ability to re‐use, integrate, or add to existing data sets can significantly speed up discoveries. The curation of digital data assets is critical for all researchers. Digital curation involves annotating, managing, and preserving digital assets. As the costs of managing digital data continue to climb, there is an increasing realization that the curation processes for biomedical research data must become more efficient and more effective. The generation and curation of biomedical data sets can be viewed as a single process involving people at different steps and levels, including scientists at all career levels, technical support staff, IT specialists, curators, and librarians.^[^ ^36^ ^]^ Therefore, there is an urgent need for advanced methods and tools to monitor and evaluate the accuracy, integrity, quality, and efficiency of the curation process for the entire data lifecycle.

## 3 EMR for Precision Medicine

EMRs are an essential component of precision medicine because they provide a comprehensive view of a patient's health data, including their medical history, test results, and treatments. This information can be used to develop personalized treatment plans that are tailored to the patient's unique genetic makeup, environment, and lifestyle.^[^ ^37^, ^38^, ^39^ ^]^ By analyzing this data, clinical decision support systems can help healthcare providers make more informed and personalized treatment decisions.

The rapid expansion of EMR data clinical implementation, driven by recent federal provisions in the HITECH Act,^[^ ^40^, ^41^ ^]^ has led to an unprecedented increase in the availability of extensive, dense, and longitudinal clinical datasets.^[^ ^40^, ^42^ ^]^ Significant efforts are already underway to link EMR data across institutions to facilitate clinical and translational research, such as studying disease onset and treatment outcomes.^[^ ^43^, ^44^ ^]^ EMR data plays a crucial role in the newly developed concept of the Learning Healthcare System (LHS).^[^ ^45^ ^]^ LHS aims to efficiently convert data about care and operations into knowledge, which is further translated into evidence‐based clinical practice and health system change. However, challenges exist when utilizing EMR data for research and operation. The heterogeneity and complex nature of EMR data make it difficult to be used directly for analysis. First, approximately 80% of EMR data (and Big Data in general) consists of unstructured data. The same clinical information can be expressed in different formats with inherent ambiguity. Second, EMR systems are highly diverse by nature.^[^ ^46^ ^]^ Relevant data could be scattered among different documents, databases, and registries in different formats. After data preparation, data mapping, and data normalization, the data can be integrated into a single repository or database. This involves developing an integration layer that enables data from different sources to be harmonized and combined based on common data elements. This can be achieved using a variety of techniques, including data warehousing, federated databases, and virtual databases.^[^ ^47^ ^]^ Before EMR data can be used for direct analysis, intensive manual data extraction and normalization processes are required, which can become a bottleneck for the secondary use of EMR data. Fortunately, recent advances in informatics research have provided alternative solutions to automate data extraction and normalization. Natural language processing technologies for extracting structured clinical data from the unstructured narrative text have shown promising results.^[^ ^48^, ^49^, ^50^ ^]^ In addition, ontology‐based approaches have also been widely applied to EMR data. For example, the Strategic Health IT Advanced Research Project for Normalization, funded by the National Coordinator for Health Information Technology, aims to develop a framework of open‐source services which can be dynamically configured to convert EMR data into standard comparable information, so as to facilitate various analysis of large‐scale data.^[^ ^51^, ^52^ ^]^

Despite the success of biomedical informatics research, current informatics tools still have very limited capacity for handling Big Data. For example, MetaMap,^[^ ^53^ ^]^ a widely used biomedical natural language processing system developed at the National Library of Medicine, takes ≈15 days to process 20 million biomedical abstracts, even when using a powerful server enabling parallel processing. EMR systems in large academic centers have accumulated decades of data, often from several million patients, each with potentially thousands of clinical records. To achieve the goal of the Learning Healthcare System, such large‐scale EMR data must be manipulated in real‐time. However, current biomedical informatics software are facing challenges in storing, querying, and analyzing such large‐scale EMR data. Therefore, there is an urgent need to introduce parallel computing frameworks for big data, such as Apache Spark^[^ ^2^ ^]^ and Hadoop,^[^ ^54^ ^]^ into the biomedical domain, thus enabling existing informatics tools to handle EMR data. By relying on the Hadoop distributed computing platform, massive EMR data can be easily processed and developed. In order to bridge the gap between raw EMR data and the need for ontological concept‐based Big Data integration and harmonization, new methods and software platforms are developed to allow parallel computing of clinical data in HER. The topics include developing novel natural language processing‐ and ontology‐based approaches to extract and normalize clinical data in EMR, and then deliver them in standard ontological formats; and developing an open‐source distributed parallel framework to speed clinical data processing and retrieval. As a platform with strong advantages in unstructured data processing, Hadoop^[^ ^1^, ^54^ ^]^ can play a key role in more medical big data application scenarios in the future. For example, Hadoop can help doctors to formulate personalized treatment plans for patients, assist in diagnosis, monitor patients' vital characteristics, etc.

### 3.1 EMR and Precision Medical Clinical Decision

Clinical decision support is the core value of EMR data.

#### 3.1.1 Clinical Decision Support System

One example of an EMR‐based clinical decision support system for precision medicine is Watson for Oncology,^[^ ^55^, ^56^ ^]^ developed by IBM. Watson for Oncology is a cognitive computing system that uses natural language processing and machine learning algorithms to analyze patient data and provide personalized treatment recommendations for cancer patients.^[^ ^57^, ^58^ ^]^ The system integrates data from EMRs, genomic data, and medical literature to provide evidence‐based treatment recommendations that are tailored to the patient's individual needs.

#### 3.1.2 Drug–Drug Interactions

Electronic medical records can identify potential drug–drug interactions, alerting physicians to potential risks of drug combinations for patients.^[^ ^59^, ^60^, ^61^ ^]^ This information can be used to tailor treatment plans to avoid adverse effects and optimize therapy.

#### 3.1.3 Personalized Medicine

Electronic medical records can help identify genetic variations that can influence drug metabolism and response. This information can be used to personalize medication regimens, improve efficacy, and reduce adverse reactions.^[^ ^13^ ^]^

#### 3.1.4 Health Care Management

A classic application of EMR data in precision medicine is for better healthcare management of patients. By designing an appropriate artificial intelligence (AI) diagnostic model, it is possible to obtain diagnostic results that are practically as accurate as or even better than those of clinicians. Although the clinical diagnosis by AI cannot guarantee completely correct results, nor can it completely replace clinicians, for a considerable number of diseases, such as pneumonia, meningitis, sinusitis, chickenpox, etc., its accuracy rate can reach more than 90%, and even exceed the detection level of doctors. For example, Brown et al.,^[^ ^62^ ^]^ proposed an algorithm for the automatic diagnosis of retinopathy based on deep learning. They used a dataset with 5511 retinal photos to train a deep convolutional neural network and achieved diagnosis accuracy equivalent to or even better than that of human experts. Adem et al.,^[^ ^63^ ^]^ designed a SoftMax classification model using a stacked autoencoder method for the automatic diagnosis of cervical cancer. The model was trained and tested on the dataset containing 668 samples and 30 attributes from the UCI database, achieving a classification accuracy of 97.8%.

#### 3.1.5 Risk Analysis

A risk assessment is the process of identifying potential hazards and analyzing possible situations when hazards occur.^[^ ^64^ ^]^ In clinical applications, the risk factor is associated with an increased risk of diseases or infections. Linear regression,^[^ ^65^ ^]^ logistic regression,^[^ ^66^ ^]^ and Cox regression^[^ ^67^ ^]^ are often used for risk analysis to evaluate the importance of risk factors. For example, based on EMR data, many scholars have established different risk prediction models to predict the readmission risk of patients.^[^ ^68^, ^69^ ^]^ Mirzaei^[^ ^70^ ^]^ compared and evaluated the agreement between cardiovascular disease (CVD) predictions using the Iran Package of Essential Non‐communicable Disease and the Framingham risk score to estimate the risk of CVD. EMR‐based risk analysis overcomes the limitations of traditional medical statistical methods, provides a new data source for disease risk assessment, and compensates for the shortcomings of traditional statistical methods in model construction.

### 3.2 EMR and Precision Medical Quality Management

Medical quality is a standard for measuring the professional competence of medical workers, as well as the overall strength of medical institutions and the treatment outcomes for specific conditions. Traditional medical quality evaluation methods are usually based on expert judgment or peer review, which are highly subjective and cannot objectively reflect the true level of medical quality. The modern clinical medical evaluation system is based on EMR. With the wide application of EMR, the diagnosis and treatment processes of various diseases have been completely recorded and stored, providing a good basis for medical quality evaluation. The advantage of the EMR system is its ability to integrate patient information scattered across different medical information systems into a unified whole. This allows data analysts to quickly access and analyze various patient data from different sources, facilitating more effective medical quality evaluations. For example, based on the EMRs and diabetes registry data, Arnold et al.^[^ ^71^ ^]^ examined the relationship between timely treatment intensification and the control of hemoglobin A1C levels as quality‐of‐care performance measures among patients with uncontrolled type 2 diabetes. To assess the quality of antibiotic use, Broek^[^ ^72^ ^]^ used EMR data from three hospitals to evaluate the appropriateness of antibiotic prescriptions, indirectly highlighting the key role of EMRs in various quality assessments within and between hospitals. Furthermore, to further confirm the significance of EMRs in medical quality, Lin^[^ ^73^ ^]^ examined the relationship between the degree of EMR adoption and patient outcomes. They divided the levels of EMR adoption degree into three groups, and designed three primary healthcare quality indicators, including inpatient mortality, readmission within 14 days, and 48‐h postoperative mortality. Finally, it was proved that full EMR adoption could improve the healthcare quality of treating severely ill patients.

EMR can also promote and improve compliance with clinical practice guidelines for patient management. Clinical guidelines are a medical standardization based on “evidence‐based medicine” that helps doctors provide appropriate medical and healthcare services under specific circumstances. The clinical practice based on EMRs plays a significant role in promoting traditional clinical guidelines. Analyzing patient data from various sources forms a knowledge base, offering a new foundation for the enhancement of clinical guidelines or clinical pathways. Sharing knowledge through diagnosis and treatment behaviors and the EMR system contributes to achieving safer and more efficient healthcare services. Terasaki et al.,^[^ ^74^ ^]^ discussed the role of the structured approach of EMRs in outpatient management of chronic obstructive pulmonary disease (COPD). Through experimental comparative analysis, they found that after implementing EMR intervention, the diagnostic screening indicators of some COPD patients showed significant improvement. Jessica et al.,^[^ ^43^ ^]^ also studied a clinical pathway for obesity hypoventilation syndrome (OHS) through EMR intervention. By comparing and analyzing the success rate of OHS patients treated with BiPAP before and after EMR intervention, it was confirmed that EMR is possible to help promote and improve BiPAP treatment.

However, there are also challenges associated with using EMRs for the applications of precision medicine. One challenge is data quality, as EMRs may contain errors or incomplete information that can affect the accuracy of clinical decision support systems. Additionally, the use of EMRs for precision medicine raises privacy and security concerns, as the data may contain sensitive information about the patient's genetic makeup and health status.^[^ ^75^ ^]^

Some applications of EMR in precision medicine are listed in **Table** **1**.

**Table 1:** Some Applications of EMR in Precision Medicine.

Works	Applications	Descriptions
Watson for Oncology^[^ ^55^, ^56^ ^]^	Clinical Decision Support System	A cognitive computing system that analyzes cancer patient data to provide personalized treatment recommendations.
DPDDI^[^ ^59^ ^]^	Drug‐Drug Interactions	A deep predictor for drug‐drug interactions.
AI diagnostic models^[^ ^62^, ^63^ ^]^	Health Care Management	Disease Diagnosis Algorithm Based on Deep Learning.
Cardiovascular disease prediction algorithms^[^ ^70^ ^]^	Risk Analysis	Estimate the risk of CVD.
Arnold et al.,^[^ ^71^ ^]^	Medical quality management	Quality‐of‐care performance measures among patients with uncontrolled type 2 diabetes.
Broek^[^ ^72^ ^]^	Medical quality management	Evaluate the appropriateness of antibiotic prescriptions.
Terasaki et al.,^[^ ^74^ ^]^	Medical quality management	Outpatient management of chronic obstructive pulmonary disease (COPD).
Jessica et al.,^[^ ^43^ ^]^	Medical quality management	Study the clinical pathway for obesity hypoventilation syndrome (OHS) through EMR intervention.

## 4 Biomedical Imaging Informatics for Translational Research and Clinical Diagnosis and Treatment

Biomedical imaging informatics is closely related to precision medicine and plays an important role in the practice of precision medicine. Biomedical imaging informatics provides valuable imaging data analysis and interpretation tools to support the implementation of precision medicine. It enables personalized diagnosis, treatment guidance, biomarker identification, treatment monitoring, image‐guided interventions, and so on, ultimately leading to more precise and effective patient care.

### 4.1 Radiomics and Radiogenomics

In contrast to the traditional practice of using medical images solely for visual interpretation, AI has made remarkable progress in image‐recognition tasks, particularly in recognizing complex patterns in imaging data and providing quantitative assessments in an automated fashion. The rapid development of radiogenomics holds great promise for various malignancies, and represents the “evolution of radiology‐pathology correlation from the anatomical‐histological level to the genetic level”.^[^ ^76^ ^]^ To enhance clinical decision‐making, radiomics focuses on extraction and analyzing quantitative imaging features of intratumoral and intertumoral heterogeneity, providing diagnostic, prognostic, and predictive data in a non‐invasive and repeatable manner. The explosion of medical imaging data creates an ideal environment for machine‐learning and data‐based science. Radiomics‐based decision‐support systems for precision diagnosis and treatment can be powerful tools in modern medicine.^[^ ^77^ ^]^ The workflow of Radiomics and Radiogenomics typically includes image acquisition, ROI segmentation, feature extraction, and finally analysis. Recently, user‐friendly and open‐access tools, such as PyRadiomics, ONCOradiomics, LIFEx, 3DSlicer, MIM, TexRAD, MaZda, and CGITA, can assist in extracting radiomics features from medical images.^[^ ^78^ ^]^ However, the workflows of Radiomics and Radiogenomics involve a significant amount of manual labor, incurring high costs. The emergence of AI has led to a seamless imaging workflow by increasing efficiency and reducing errors.

### 4.2 Medical Imaging Analysis

**Figure 1:**
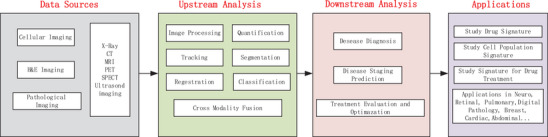
Medical Image Analysis Routines.

Medical imaging includes cellular imaging, H&E, and other pathological imaging, as well as X‐Ray, CT, MRI, PET, SPECT, and Ultrasound imaging. Traditional image analysis involves fundamental steps such as preprocessing, registration, alignment, segmentation, quantification, tracking, and classification, as illustrated in **Figure** **1**. Recent advancements in deep learning circumvent some of these steps by directly inputting raw imaging data into various networks and outputting classification labels. The various processing steps of classic machine learning methods, which involve handcrafted features as seen in radiomics, are replaced by deep medical image feature extraction or an end‐to‐end learning approach of deep learning methods.^[^ ^79^ ^]^

In recent years, a trend in both research and clinical applications has been the exploration of different kinds of deep neural network architectures to make them applicable to various medical images and different application scenarios.^[^ ^80^ ^]^ Different variations of convolutional networks^[^ ^81^, ^82^ ^]^ and transformer‐based networks^[^ ^83^, ^84^ ^]^ are widely used methodologies for tasks such as image classification, object detection, segmentation, registration, and so on. Deep learning algorithms have achieved state‐of‐the‐art performance while eliminating the need for manual feature extraction algorithms.^[^ ^85^, ^86^, ^87^, ^88^ ^]^ Many promising applications have been reported in neuro,^[^ ^89^ ^]^ retinal,^[^ ^90^ ^]^ pulmonary,^[^ ^91^ ^]^ digital pathology,^[^ ^92^ ^]^ breast,^[^ ^93^ ^]^ cardiac,^[^ ^94^ ^]^ abdominal,^[^ ^95^ ^]^ and musculoskeletal,^[^ ^96^ ^]^ and so on. For cellular imaging analysis, AI technologies are used to study drug signatures by examining cell phenotypes and cell phase changes before and after treatment.^[^ ^97^, ^98^, ^99^, ^100^ ^]^ In this article,^[^ ^101^ ^]^ both the mathematical mechanics and the programming frameworks of cellular image analysis are reviewed. For H&E and other pathological image analyses, cell population signatures are explored in response to drug treatment.^[^ ^98^, ^102^ ^]^

Another research hot topic is multi‐modal medical image fusion by merging multiple images from single or multiple imaging modalities to improve the imaging quality while preserving the specific features.^[^ ^103^, ^104^ ^]^ Although most studies are based on single‐modality data, cross‐modality fusion techniques are able to further improve the performance of downstream tasks in medical image analysis if multiple modalities of image resources are properly fused.^[^ ^105^, ^106^ ^]^

Though the deep learning‐based methods obtain promising results in research environments, they still face challenging issues in clinic application environments, such as model robustness, model performance, model interpretability, and data collecting and sharing.

The model robustness refers to the performance using the trained model to test on real medical images in clinic may have a gap between the validation datasets. Collecting large balanced diverse data is one good practice to improve model robustness. And recent papers argue that the quality of training data is as important as the data size. However, medical images are usually unbalanced and difficult to collect, then, data augmentation methods can be used to improve model performance. In the study by Frid‐Adar et al.,^[^ ^107^ ^]^ Generative Adversarial Networks (GANs) were used to generate synthetic medical images, and the performance of CNN for medical image classification improved using synthetic data together with the real data. Transfer learning is another widely used strategy. Instead of only training a network with a limited amount of medical image data, transfer learning first trains the network with other larger source data sets, creating a more robust model. In the study by Cheplygina,^[^ ^108^ ^]^ the classification performance in lung CT images was improved using transfer learning strategy by using additional cat images to pretrain the model. Domain adaptation techniques^[^ ^109^ ^]^ were used to make model's performance robust, and self‐supervised deep learning techniques^[^ ^110^ ^]^ were used to ease the requirement of labor‐consuming data labeling works.

Another challenging issue of deep neural network is weak interpretation. As the deep learning methods are usually in end‐to‐end training form, which makes the model hard to explain and accepted by clinic doctors. Some promising techniques have been proposed to solve these challenging issues, such as SHAP value techniques to make deep learning models explainable.^[^ ^111^ ^]^ In Zhang et al.,^[^ ^112^ ^]^ a semantically and visually interpretable medical image diagnosis network was proposed by exploring multimodal mapping between medical images and diagnostic reports to provide justifications of the network diagnosis process.

Some topics of medical imaging analysis are listed in **Table** **2**.

**Table 2:** Some Topics of Medical Imaging Analysis.

Applications	Topics	Data Sources
Neuro,^[^ ^89^ ^]^ retinal,^[^ ^90^ ^]^ pulmonary,^[^ ^91^ ^]^ digital pathology,^[^ ^92^ ^]^ breast,^[^ ^93^ ^]^ cardiac,^[^ ^94^ ^]^ abdominal,^[^ ^95^ ^]^ and musculoskeletal,^[^ ^96^ ^]^	Deep neural network architectures	MRI, funduscopic images, lung image, digitizing whole‐slide images, X‐Ray, cardiovascular images, body composition images, musculoskeletal imaging,
Clinical treatment analysis,^[^ ^106^ ^]^	Multi‐modal medical image fusion	CT, MRI, PET.
GANs,^[^ ^107^ ^]^ Transfer learning,^[^ ^108^ ^]^ Domain adaptation techniques,^[^ ^109^ ^]^ Domain adaptation techniques	Data augmentation	CT, MRI.

## 5 AI‐Aided Surgical Design and Surgery Optimization

By integrating AI technology into surgical practices, healthcare providers can deliver more precise, efficient, and tailored surgical interventions, ultimately enhancing patient outcomes in the era of precision medicine. AI‐Aided Surgery Design and Surgery Optimization includes the following major topics: 1) Predictive modeling and AI using biomedical big data and surgical planning/design and surgical optimization; 2) AI‐aided surgery training and surgery evaluation; and 3) AI‐aided surgery assistance.

### 5.1 Surgical Planning/Design/Optimization

The latest direction in translational research is integrating imaging and EMR to aid precision surgery design and optimize medical devices. Here, we focus on the following topics, including i) medical image segmentation, registration, quantification, and patient stratification based on image features, demographics, diagnostic features, and physical features, ii) simulation of biomechanical properties of different tissues and organs in patients with different behaviors using finite elemental models, iii) optimization of medical device parameters at the system level for clinical outcomes and precision surgery based on clinical factors, image features, and biomechanical features using machine learning approaches, and iv) clinical applications with optimized medical devices and precision therapy.

Various approaches have been proposed i) for dental clinicians to simulate the facial soft‐tissue‐changes resulting from skeletal reconstruction (eFace project);^[^ ^113^ ^]^ ii) for osteoarthritis clinicians to simulate the articular osteochondral tissue behavior affected by femoral angle change after unicompartmental knee arthroplasty (eOA system);^[^ ^114^, ^115^ ^]^ iii) for pediatric surgeons to make optimal surgery design for craniosynostosis (CSO) patients;^[^ ^116^ ^]^ iv) for surgeons to optimize the mandibulectomy design and free flap reconstruction (a novel eMandible system);^[^ ^117^ ^]^ v) for cardiac surgeons to predict the risk of critical complications, including complete atrioventricular block (AVB) and paravalvular aortic regurgitation (AR) after transcatheter aortic valve replacement (TAVR), and optimization of TAVR device for surgery (eValve project);^[^ ^118^ ^]^ and vi) for pancreas clinicians to predict timing of surgical intervention using recurrent neural network for necrotizing pancreatitis.^[^ ^119^ ^]^ In Knoops et al.,^[^ ^120^ ^]^ a large‐scale clinical 3D morphable model, trained with 4261 faces of healthy volunteers and orthognathic (jaw) surgery patients, demonstrated how deep learning model could fully automatically aid diagnosis and provided patient‐specific treatment plans from a 3D scan alone, to help efficient clinical decision making and improve clinical understanding of face shape as a marker for primary and secondary surgery. Here, are just a few examples. These studies can improve clinical outcomes substantially.

### 5.2 Big Data‐Driven AI‐Aided Surgery Training, Surgery Evaluation

Virtual reality has been proven effective for both resident training and preoperative planning. Big data‐driven AI‐aided virtual reality surgery training and preoperative planning have been successfully applied in orthopedic surgery, maxillofacial surgery, and neurosurgery.^[^ ^121^, ^122^ ^]^ In Zhou et al.,^[^ ^123^ ^]^ AR/VR technology builds an anatomical environment combining virtual and real by fusing the clinical imaging data and information, which is helpful to human anatomy surgery teaching. During the training, large amount of diverse data, such as object poses and segmentation maps, can be generated for validating and training downstream computer vision algorithms.

With the help of augmented reality, surgeons could carry out procedures faster and more accurately, improving overall safety. With the rapid development of AI, videos of surgical procedures can be automatically analyzed. Not only can the instruments be recognized, but also the anatomical structure, surgery actions, and surgery stages using surgical process models. The detailed information can be used to assess surgical procedure quality. If some unwanted situation appears, the system can raise warning signs and tips for preventing surgery accidents. Some successful applications have been reported.^[^ ^124^, ^125^ ^]^

### 5.3 Big Data‐Driven AI‐Aided Surgery assistance

Big data is one key element to enable real robotic surgery to recognize, process, predict and ultimately execute tasks, either under supervision or unsupervised from human control rather than being only a “tele‐manipulator”. The importance of collecting such masses of data and sharing information in an honest, transparent, and collaborative manner, and organizing originally unstructured, heterogeneous data into “usable” machine learning data is emphasized.^[^ ^126^ ^]^ How to effectively incorporate information obtained from AI algorithms into real‐time surgical procedures in order to provide more helpful guidance, simplify complex surgical manipulations, and issue urgent warnings in case of unexpected situations.^[^ ^127^ ^]^

## 6 Applications of Omics Data for Precision Medicine in Bioinformatics

Omics data provides valuable molecular insights into diseases, patients, and treatment response, facilitating the practice of precision medicine. It enables molecular characterization, identification of predictive biomarkers, development of personalized treatment strategies, and disease monitoring. By integrating omics data into clinical decision‐making, healthcare providers can deliver targeted and individualized interventions to improve patient outcomes.

### 6.1 Omics Data, Tools, and Resources

**Figure 2:**
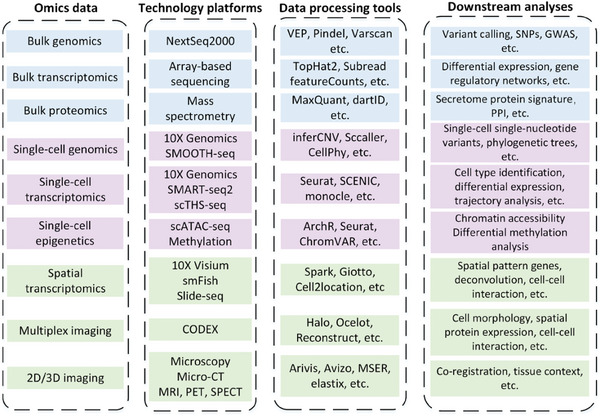
Summary of omics data, available computational tools, and downstream analyses based on omics data.

With the development of sequencing technology, high throughput omics data such as genomics, transcriptomics, epigenomics, proteomics, metabolomics, and microbiome data are exploding. These data have been used extensively over the past 20 years to understand disease mechanisms and treatments. To gain biological insights from omics data, one needs to be familiar with complex computational techniques and available data resources. Here, we briefly introduce recent advances in omics data integration and analyses as summarized in the automated pipelines shown in **Figure** **2**.

Bulk sequencing is the standard tool to generate data at the individual level. Bulk DNA sequencing data are used for variant calling with standard tools such as MuSE, CaVEMan, and GATK.^[^ ^128^, ^129^, ^130^ ^]^ Bulk RNA sequencing pipelines are based on TopHat2, MapSplice, and Cufflinks for transcript alignment and annotation.^[^ ^131^, ^132^, ^133^ ^]^ It is possible to understand gene interaction networks and decipher complex gene regulatory networks by performing standard methods such as clustering and correlation using bulk sequencing data.

Single‐cell/single‐nucleic sequencing (e.g., sc/snRNA‐seq and sc/snDNA‐seq) refers to the sequencing of transcriptome or genome in individual cells. For sc/snRNA‐seq data, we can use Harmony to remove batch effects and Seurat to filter and cluster the cells.^[^ ^134^, ^135^ ^]^ Cell clusters can be annotated with cell types and states based on the expression of marker gene sets. Other analyses include differential expression analysis, cell‐cell interaction (CCI) analysis, and TF regulatory network analysis. Several tools can be used for these analyses, such as limma, DESeq2, CellphoneDB, CellChat, and SCENIC.^[^ ^136^, ^137^, ^138^, ^139^, ^140^ ^]^ Results can be scanned for over/under‐expressed genes, ligand‐receptor interaction, and cell‐specific enriched motifs and transcript factors.

Spatial transcriptomics (ST) is a set of techniques that assign cell types to their locations in histological sections.^[^ ^141^ ^]^ This method provides a map by combining transcriptomic information and spatial information innovatively. ST can be integrated with sc/snRNA‐seq using Seurat to spatially resolve single‐cell expression data via building and quantifying barcoded spot‐to‐spot or cell‐to‐cell interaction matrices. Accurate deconvolution of the observed gene expressions at each spot and recovery of cell constitutions can be performed with approaches such as cell2location.^[^ ^142^ ^]^ SpatialDE, Trendsceek, SVCA, and SPARK are used to search for spatial patterns in gene expression.^[^ ^143^, ^144^, ^145^, ^146^ ^]^ Furthermore, cell–cell interaction (CCI) is defined as cells communicating with each other in response to changes in their microenvironment, which is important in many biological processes. Dissecting CCIs will provide a better understanding of how cells cooperate in response to external perturbations that lead to corresponding functional and pathological changes in the tissues. For example, SpaOTsc can measure CCIs based on three major optimization terms.^[^ ^147^ ^]^

3D genome architecture has shown rapid progress as research continues to advance single‐cell technologies for analyzing the whole genome, DNA methylation, and chromatin accessibility. Moreover, high‐throughput sequencing could also be used in other fields, such as microbiology. Recently, Zheng et al. presented microbe‐seq, which can generate genomes of individual microbes from complex microbial communities in the human gut.^[^ ^148^ ^]^ Omics data, combined with extensive tools and pipelines, allows multi‐level applications on the molecular mechanism of health and diseases.

Additionally, a wealth of data resources strongly supports diverse research communities to promote personalized medicine. We summarized bioinformatics resources and databases into five categories, including cell line‐based database, sample‐based database, single cell‐based database, signature‐based database, and functional annotation‐based database. Here, some databases and their brief descriptions are listed in **Table** **3**. These resources are vitally important to explore the potential mechanism, identify drug targets, and validate biomarkers. For example, drug targets can be identified by using The Library of Integrated Network‐Based Cellular Signatures (LINCS) and Genomics of Drug Sensitivity in Cancer (GDSC). Related functions of certain biomarkers can be found in signature‐based databases and functional annotation‐based databases. TCGA and GEO datasets can be used to predict drug response in patients. Moreover, due to the development of single‐cell sequencing and spatial transcriptomics techniques, extensive databases storing large amounts of cells from multiple tissues or disease samples may further promote disease signature extraction and personalized medicine.

**Table 3:** Summary of Several Types of Bioinformatics Resources and Databases.

Resources	Data type	Description
Cell line‐based database		
LINCS^[^ ^149^ ^]^	Omics data for cell lines	LINCS provides observation of how and when a cell's phenotype is altered by specific stressors can provide clues about the underlying mechanisms involved in perturbation and, ultimately, disease.
CCLE^[^ ^150^ ^]^	Omics data for cancer cell lines	CCLE provides the basal genetic background for the LINCS drug response datasets.
GDSC^[^ ^151^ ^]^	Drug response data for cell lines	GDSC provides a large‐scale drug screen incorporating detailed genomic analyses for cancer cell lines to systematically identify drug response biomarkers.
Sample‐based database		
UKBiobank^[^ ^152^ ^]^	Clinical data, genetic data, imaging data	UKBiobank provides a long‐term study resource with health and genetic information for 500000 volunteers in the UK, enrolled at ages from 40 to 69.
TCGA	Omics data and clinical data for cancer patients	TCGA is a project to catalogue the genetic mutations responsible for cancer using genome sequencing and bioinformatics.
GEO^[^ ^153^ ^]^	Omics data and clinical data uploaded by researchers	GEO included multiple omics datasets uploaded by researchers, which good for disease signature extraction and improved personalized medicine.
Oncomine^[^ ^154^ ^]^	Microarray data for cancer patients.	Oncomine provides gene expression data for advancing cancer research and improving cancer patient outcomes in precision oncology.
ArrayExpress^[^ ^155^ ^]^	Omics data and clinical data uploaded by researchers	ArrayExpress is one of the repositories recommended by major scientific journals to archive functional genomics data from microarray and sequencing platforms to support reproducible research.
Single cell‐based database		
scRNASeqDB^[^ ^156^ ^]^	scRNA data	scRNASeqDB contains 36 human single cell gene expression data sets, involving 8910 cells from 174 cell groups.
Human Cell Atlas (HCA)^[^ ^157^ ^]^	scRNA data	HCA provides an open comprehensive reference map of the molecular state of cells in multiple healthy human tissues.
CancerSEA^[^ ^158^ ^]^	scRNA data for multiple cancer types	CancerSEA aims to comprehensively explore 14 functional states of cancer cells at the single‐cell level.
Single Cell Portal	scRNA data uploaded by researchers	Single Cell Portal provides visualization, download and analysis of scRNA datasets for multiple tissues, diseases and species.
PanglaoDB^[^ ^159^ ^]^	scRNA data from mouse and human	PanglaoDB provides multiple datasets collected from mouse and human samples. The goal of PanglaoDB is to provide easy access and exploration of scRNA data.
SpatialDB^[^ ^160^ ^]^	ST data	SpatialDB provides manually curated resource of spatially resolved transcriptomes. It also provides visualization and comparison of ST data.
SC2disease^[^ ^160^ ^]^	scRNA data for multiple diseases	SC2disease is a comprehensive resource for documenting cell‐type‐specific genes of human diseases.
Aging Atlas^[^ ^161^ ^]^	scRNA data from aging studies	Aging Atlas provides user‐friendly functionalities to explore and download age‐related changes in gene expression.
Signature‐based database		
MSigDB^[^ ^162^ ^]^	Annotated gene sets	MSigDB provides more than 10000 gene sets by using gene set enrichment analysis.
KEGG^[^ ^163^ ^]^	Networks of genes and molecules	KEGG is a database resource for understanding high‐level functions and utilities of the biological system, such as the cell, the organism and the ecosystem.
UCSC genome browser^[^ ^164^ ^]^	Genome sequence data	UCSC provides an interactive website offering access to genome sequence data from a variety of vertebrate and invertebrate species and major model organisms, integrated with a large collection of aligned annotations.
SMPDB^[^ ^165^ ^]^	Small molecule pathways	SMPDB is an interactive, visual database containing more than 30 000 small molecule pathways found in humans only.^[^ ^166^ ^]^
JASPER	Transcription factor (TF) binding profiles	JASPER contains a curated, non‐redundant set of profiles, derived from published collections of experimentally defined transcription factor binding sites for eukaryotes.
Functional annotation‐based database		
FusionGDB^[^ ^167^ ^]^	Fusion Gene annotation	FusionGDB provide a resource or reference for functional annotation of fusion genes in cancer for better therapeutic targets.
miRDB^[^ ^168^ ^]^	miRNA targets annotation	miRDB is an online database for miRNA target prediction and functional annotations.
MethyCancer^[^ ^169^ ^]^	DNA methylation annotation	MethyCancer contains both highly integrated data of DNA methylation, cancer‐related gene, mutation and cancer information from public resources, and the CpG Island (CGI) clones derived from large‐scale sequencing.
Lncbase^[^ ^166^ ^]^	lncRNA and miRNA annotation	Lncbase contains ∼240000 unique tissue and cell‐type specific miRNA‐lncRNA interactions.

### 6.2 Opportunities for Omics Data and AI in Advancing Precision Medicine

#### 6.2.1 Deciphering Cellular and Genomic Heterogeneity in Diseases

Classical genetics divided diseases into two categories, including Mendelian diseases and complex traits and diseases. Mendelian diseases are attributed to single gene mutation with a simple pattern of inheritance, while complex traits and diseases are due to multiple gene mutations, alterations, and interactions.^[^ ^170^ ^]^ These factors directly pose greater challenges for understanding complex disease traits. However, as these are far more common than Mendelian diseases, understanding the complex traits of diseases has more socioeconomic impact.^[^ ^171^ ^]^

Heterogeneity is one of the major obstacles to understanding the aetiologies of complex traits and diseases. For example, cancer is a highly heterogeneous disease with multiple clonal and subclonal populations leading to tumor evolution and drug resistance, which push bulk sequencing technologies to their limits. Currently, single‐cell and ST technologies and AI advance the knowledge of heterogeneity and diversity of cell populations. Several tools can be used to estimate copy number aberrations and clonal deconstruction of cancer evolution, such as inferCNV, SCONCE, SCITE, and LACE.^[^ ^172^, ^173^, ^174^, ^175^ ^]^ One of the key questions closely related to therapy is which cells are more likely to metastasize and whether the metastases are seeded early in tumor evolution progression or isolated from later cells. Currently, large omics genomic data provide the possibility of distinguishing phylogenetic patterns between different cell populations. These may help monitor cancer progression and identify combination treatment strategies.

#### 6.2.2 Improving Immune Therapy by Omics Data

The last decade has witnessed tremendous advances in the clinical treatment of cancer patients through immunotherapy. Immune‐checkpoint inhibitors (ICIs), such as PD1, PDL1, and CTLA‐4, have been widely used in many cancer types. These ICIs have increased the patient's survival and are approved by Food and Drug Administration (FDA).^[^ ^176^ ^]^ However, the major limitations of ICIs include only a minority of patients responding to immunotherapy and immune‐related adverse events (IRAEs). Early treatment response data and IRAEs came from case reports or case series, but a comprehensive understanding of toxicities and prediction of treatment response requires many patients and sample data. Here, we listed some omics resources and medical AI algorithms that can be applied to accelerate clinical and translational research on immunotherapy. ClinicalTrials.gov records more than 1000 published clinical trials that tested anti‐PD‐1/PD‐L1 antibodies in >100 000 patients with cancer between 2010 and 2020.^[^ ^177^ ^]^ FDA Adverse Event Reporting System (FAERS) is a public database that stores spontaneous reports on IRAEs related to FDA‐approved products, which has been widely used to study drug response and IRAEs.

Additionally, the rapid generation of genomic research and bioinformatics resource brings an enormous amount of multi‐omics data. Especially, immunomics data can provide information on the immune system at the molecular and cellular levels to help identify drug targets. For example, Machine learning algorithms can be developed to translate T cell/B cell receptor sequences into antigens that they can target. Single‐cell sequencing of immune cells can provide a tumor‐specific immune microenvironment system that can enlighten the identification of immune therapy targets. Based on large‐scale omics data, extensive algorithms have been developed to help gain a more comprehensive understanding of immune therapy in diseases. For example, NetBio was developed to predict ICI treatment biomarkers and treatment response by using L2 regularized logistic regression.^[^ ^178^ ^]^ Prediction tools such as pVAC‐seq, TSNAD, Neopepsee, and INTEGRATE‐neo have been developed to prioritize tumor‐specific candidate neoantigens.^[^ ^179^, ^180^, ^181^, ^182^ ^]^

#### 6.2.3 Uncovering Drug Repurposing Strategies in Existing Omics Data

**Figure 3:**
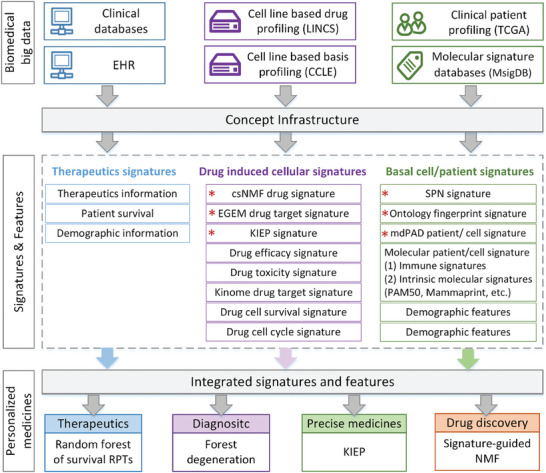
Summary of Signatures: Sources, types, and applications. Biomedical big data can be collected from public databases and EHR. Signatures can be extracted from biomedical big data by using computational tools. These signatures can be used on multiple aspects such as diagnosis, therapy strategies and drug discovery. Red stars mean the structured signatures. HER, Electronic health record; csNMF, systematic compound signature discovery pipeline; EGEM, Enrichment of gene effects of a molecule; SPN, Segment polarity network; KIEP, Kinase inhibitor effect prediction.

Drug discovery is a time‐consuming, laborious, costly, and high‐risk process. The research and development of a new drug may take 10–15 years. Development of a new drug may cost more than $2 billion and the cost is increasing year by year.^[^ ^183^ ^]^ The compound databases have facilitated numerous studies of the similarity of compounds based on various properties. Recently, drug repositioning has been an attractive approach to discovering drugs for diseases. Various data‐driven computational or analyzing methods were reported for drug repurposing. One active direction is drug repurposing between cell lines and clinical samples, like TCGA patients. Drug repurposing methods can be divided into two categories, including network‐based and structure‐based methods. For network‐based drug mining, Chyr et. al recently developed an approach named Deep Learning Optimal Transport Approach (DOTA).^[^ ^184^ ^]^ It is a novel and stable network‐based deep‐learning method for repositioning drugs for Alzheimer's disease (AD) treatment. The effect of DOTA on drug discovery advanced due to it considering drug targets, side effects, and associations with other diseases in its predictions. Structure‐based drug mining is also widely used. The core idea of gene signature‐based drug mining is that candidate drugs should restore genomic signatures that are altered by the disease compared with the controls. The major types of signatures, upstream biomedical big data resources, and downstream applications are summarized in **Figure** **3**. Several resources and databases play important roles in drug mining, such as the drug‐gene interaction database (DGIdb)^[^ ^185^ ^]^ and Open Targets database.^[^ ^185^, ^186^ ^]^ Combined with ML algorithms such as deep learning, these databases could allow the field of drug mining to flourish. An unresolved challenge in small‐molecule design is how to represent chemical structures best. There are a plethora of representations ranging from simple circular fingerprints, such as the extended‐connectivity fingerprint, to sophisticated symmetry functions. It is still unclear which structure representation is best suited for which small‐molecule design problems. Recently, DeepMind company developed Alphafold2 to predict protein structures with significantly improved accuracy.^[^ ^187^ ^]^ It will be interesting to see if the rise of ML studies in the field of cheminformatics will provide more guidance on the best choices for future structure representations.

#### 6.2.4 Pharmacokinetics‐Pharmacodynamics Models

Traditional drug development methods are time‐ and resource‐ consuming and carry a substantial risk of adverse effects. Hence, a more efficient method is urgently needed to predict pharmacology and toxicology by applying systems approaches to understanding pharmacokinetics‐pharmacodynamics (PKPD). PKPD is a wide field including topics such as nanoparticle delivery and release. Here, we briefly discuss multiscale modeling to predict drug absorption, distribution, metabolism, excretion (ADME), and drug interactions at target sites from the cellular level to system level based on multimodality imaging. One of the most critical challenges is the application of computational modeling for PKPD analysis. Pharmacokinetics models can be used to describe or predict the distribution of drugs in the body compartments like blood and heart. In contrast, pharmacodynamics models focus on the time course of drug effects at the site of action. Both types of models are necessary in contemporary drug research. However, if they were viewed as two separate processes, they may not fully facilitate drug research. Some pharmaceutical companies like SimCYP and Entelos have been working on a combined PKPD approach to assist drug research and development.^[^ ^188^, ^189^ ^]^ They directly use organ‐level PKPD data to predict concentration changes for a new drug. However, this traditional modeling approach fail to consider the biophysical properties and heterogeneity of different tissues and interstitial tumor space. In one of our previous models, we studied drug release at the tissue level based on the biophysical properties of interstitial tumor space.^[^ ^190^ ^]^ This work systematically integrated molecular imaging, biophysical modeling, and biological pathway modeling to study drug ADME and interactions at target sites.

In short, the heterogeneity of complex traits and diseases leads to diversity in patient data. It requires multi‐omics data at the individual level, AI techniques, and intensive analyses to understand the underlying mechanisms and advance knowledge of “precision medicine”.

## 7 Health Monitoring Data for Precision Medicine

The rapid development of sensor technology and the Internet of Things (IoT) has enabled the healthcare industry to monitor patients' physiological conditions in real‐time and remotely, providing unprecedented opportunities for personalized and precision medicine. Through sensors, various physiological parameters such as Electroencephalogram (EEG), Electrocardiogram (ECG), heart rate, blood pressure, blood glucose levels, temperature, and physical activity can be measured and recorded. These sensors can be embedded in wearable devices, medical equipment, and mobile applications to collect patients' physiological data. This data can be transmitted in real‐time to healthcare professionals or medical information systems through cloud computing and internet connectivity, providing timely health information for both doctors and patients.

Health monitoring data possesses real‐time capabilities, enabling doctors to gain more accurate insights into patients' physiological states and make rapid diagnostic and treatment decisions. By analyzing real‐time data, healthcare professionals can promptly identify potential health issues and take appropriate intervention measures. For example, to prevent driver fatigue‐related accidents, it is feasible to analyze wearable EEG signals for drowsiness detection and timely warnings, ensuring the safety of automotive driving.^[^ ^191^ ^]^

In the process of continuous monitoring and recording of patients' physiological parameters, medical records, and healthcare information, health monitoring data exhibit characteristics of long‐term time series data, posing significant challenges for data analysis and utilization. Long‐term time series data often include observations at multiple time points, making the dataset very large and complex, requiring substantial computing and storage resources for processing and storage. Furthermore, issues such as data missingness may affect the accuracy of data analysis, and sensor drift, instrument calibration issues, or environmental changes can lead to a decline in data quality and stability, necessitating data quality control and calibration. Additionally, the analysis of long‐term time series data demands more advanced data mining techniques to uncover long‐term trends, periodic patterns, anomaly events, and potential associations.^[^ ^192^, ^193^ ^]^

Health monitoring data forms the foundation for personalized medicine. First, by comprehensively analyzing a patient's health monitoring data alongside other medical data, healthcare professionals can tailor treatment plans to individual circumstances, including adjustments in medication dosages, dietary recommendations, and exercise regimens. Second, health monitoring data aids in early diagnosis and disease prediction, enabling the identification of potential health problems before symptoms manifest, facilitating timely interventions.^[^ ^194^, ^195^ ^]^ Additionally, health monitoring data empowers patients to actively engage in their own health management.^[^ ^196^ ^]^ By accessing their personal monitoring data, patients can better understand their health status and take proactive steps to improve their lifestyles and treatment adherence.

Through continuous improvements in sensor technology, data analysis methods, and privacy protection measures, we have the potential to achieve more personalized, efficient, and secure healthcare, providing better medical experiences and health management services for patients. Nevertheless, the application of health monitoring data in precision medicine comes with certain challenges. First, there are concerns about data privacy and security, as these data involve patients' health information, necessitating stringent privacy protection measures to ensure data is not accessed by unauthorized parties. Second, data accuracy and reliability are crucial issues, requiring the development of high‐quality sensors and corresponding algorithms to ensure data reliability. Third, ethical, legal, and societal issues must be closely monitored to ensure that the application of health monitoring data adheres to ethical and legal guidelines and maximizes benefits for patients.

## 8 Knowledge Graph for Precision Medicine

Precision medicine is an approach to healthcare that customizes medical treatment to an individual's specific genetic, environmental, and lifestyle factors. Knowledge graphs,^[^ ^197^ ^]^ which are visual representations of the relationships between various types of data and information, can serve precision medicine in several ways.^[^ ^198^, ^199^, ^200^ ^]^ Medical knowledge graph^[^ ^201^ ^]^ is a knowledge base, which expresses the disease information and its relationship through the nodes and edges of the graph.^[^ ^202^ ^]^ Graph‐expressed disease information allows for graph‐based methods to query and work with the medical data. In the Lexigraph, there are three types of nodes (drugs, anatomy parts, and diseases), which are connected to each other. Take diabetes for example, Diabetes Type I (node 1) and Diabetes Type II (node 2) are both types of Diabetes (node 3).

Most of the existing medicine recommendation systems are based mainly on EMRs,^[^ ^203^, ^204^ ^]^ which significantly assist doctors in making better clinical decisions. In the era of big data, although the growth speed of EMRs is fast, the content in EMRs limits a lot. Taking drug‐drug interactions for example, the EMRs cannot reflect some medical facts. Some medical knowledge graphs containing drug‐related information, such as DrugBank, may solve the shortage of EMRs.^[^ ^205^ ^]^ However, the robustness of medical knowledge graph is often affected by the incompleteness of the graphs.

To cope with these challenges, Zhang stands on advances in graph embedding learning techniques^[^ ^206^ ^]^ and proposed a novel framework called Safe Medicine Recommendation (SMR).^[^ ^207^ ^]^ First, SMR constructs a high‐quality heterogeneous graph by integrating EMRs (MIMIC‐III) and medical knowledge graphs. Then, diseases, medicines, patients, and corresponding relations are embedded into a shared lower‐dimensional space. At last, the embeddings are used to convert the medicine recommendation to a link prediction process, and the patient's diagnoses and adverse drug reactions are also considered.

With the increasing application of knowledge graphs,^[^ ^208^ ^]^ many researchers have extracted information from medical databases and then constructed heterogeneous knowledge graphs about medicines and diseases. For instance, DrugBank is a rich source of medical information, and it contains a wide range of entities (drugs, drug targets, chemistry, etc.) and relationships (enzymatic pathways, drug–drug interactions, etc.).^[^ ^205^ ^]^ The ICD‐9 ontology is a knowledge base of human diseases that can be used to classify patient diagnoses.^[^ ^209^ ^]^ Using the medical knowledge graph in the EMRs‐based medicine recommendation system^[^ ^210^ ^]^ may enable the recommendation system to provide more accurate medical services for particular patients.

Integrating EMRs and medical knowledge graphs to construct a high‐quality heterogeneous graph can expand the scope of medical recommendation. However, this task may confront the following challenges: 1) Computational Efficiency. When querying specialized medical entities and relationships, graph‐based algorithms often have limitations in portability and scalability, so it may become unfeasible when the heterogeneous medical graph reaches a considerable scale. 2) Data Incompleteness. Although medical knowledge graphs play an important role in medical recommendation systems and other applications, in fact, most of the medical knowledge graphs are generally incomplete. For example, since the DDIs are often not identified at the clinical trial phase, there is a lack of significant DDIs in DrugBank, which affects the drug recommendation. 3) Cold Start. Conventional drug recommendation systems are usually based on historical records, which is difficult to keep up with the update speed of new therapies and treatments in medical practices. The medical knowledge graphs often lack new drug and treatment information. Considering these problems, Wang et al.^[^ ^207^ ^]^ designed a new drug recommendation framework based on graph embedding technology named SMR. In the SMR framework, a joint learning algorithm is proposed to solve the cold start problem.

As described, the knowledge base contains signatures of various types and at different conceptual levels, from the low‐level molecular and cellular levels to higher levels, such as individual patients or diseases. For a given signature type, each extracted signature is essentially a set of features; a given signature can be viewed as a point in a multidimensional feature space. Different methods have been proposed for indexing in multidimensional feature spaces,^[^ ^211^, ^212^, ^213^ ^]^ and most of these methods work well for specific features, distance measures, or query types. Knowledge graphs can serve as powerful tools for advancing precision medicine by integrating multiple sources of data and information, identifying patterns and relationships between different types of data, and keeping clinicians and researchers up to date with the latest advances in the field.

Here are a few examples of knowledge graphs serving precision medicine:

Cancer Knowledge Graph: This is a comprehensive database of cancer genomics and is designed to help researchers and clinicians identify genetic mutations that are associated with specific types of cancer.^[^ ^214^, ^215^, ^216^ ^]^ The graph integrates data from multiple sources, including genomics, transcriptomics, proteomics, and metabolomics, to provide a comprehensive view of the molecular networks that underlie cancer.

Drug–Target Interaction Graph: This is a comprehensive database of drug–target interactions and is designed to help researchers and clinicians identify potential drug targets for specific diseases.^[^ ^217^, ^218^, ^219^, ^220^, ^221^ ^]^ The graph integrates data from multiple sources, including drug databases, protein databases, and gene expression databases, to provide a comprehensive view of the molecular networks that underlie drug‐target interactions.

Infectious Disease Knowledge Graph: This is a comprehensive database of infectious disease genomics and is designed to help researchers and clinicians identify genetic mutations that are associated with specific infectious diseases.^[^ ^222^, ^223^ ^]^ The graph integrates data from multiple sources, including genomics, proteomics, and clinical data, to provide a comprehensive view of the molecular networks that underlie infectious diseases.

Pharmacogenomics Knowledge Graph: This is a comprehensive database of pharmacogenomics data and is designed to help researchers and clinicians identify genetic variations that are associated with drug response.^[^ ^224^, ^225^ ^]^ The graph integrates data from multiple sources, including genomics, drug databases, and clinical data, to provide a comprehensive view of the molecular networks that underlie drug response.

Rare Disease Knowledge Graph: This is a comprehensive database of rare disease genomics and is designed to help researchers and clinicians identify genetic mutations that are associated with specific rare diseases.^[^ ^226^, ^227^ ^]^ The graph integrates data from multiple sources, including genomics, proteomics, and clinical data, to provide a comprehensive view of the molecular networks that underlie rare diseases.

## 9 Public Health Informatics

Public health informatics (PHI) is defined as the systematic application of information, computer science, and technology in public health, including surveillance, prevention, preparedness, and health promotion.^[^ ^228^, ^229^ ^]^

First, public health big data can provide the data and information needed for precision medicine. For example, public health big data can provide large‐scale population health data and information, helping healthcare institutions and researchers understand the overall health status of a population, identify differences in health status and disease trends across different populations, and develop more precise disease prevention and intervention strategies.^[^ ^230^, ^231^, ^232^ ^]^

The National COVID Cohort Collaborative (N3C)^[^ ^233^, ^234^, ^235^ ^]^ is a large‐scale, nationwide initiative to create a centralized, harmonized data platform to accelerate research on COVID‐19 and its impact on health outcomes. The N3C platform is a valuable resource for precision medicine researchers interested in studying COVID‐19.

The N3C data platform integrates clinical data from multiple sources, including electronic health records, claims data, and other COVID‐19‐related data. By integrating and harmonizing these data sources, researchers can access a large and diverse dataset that enables the exploration of questions related to COVID‐19 and its impact on health outcomes.

The N3C platform also provides access to advanced analytical tools, including machine learning algorithms, natural language processing, and data visualization tools. These tools allow researchers to extract insights from the data and generate hypotheses that can be further explored using more targeted studies.

Precision medicine researchers can use the N3C platform to study the impact of COVID‐19 on specific populations, identify potential risk factors, and develop personalized treatment strategies. For example, the N3C platform could be used to identify genetic or other biomarkers that predict COVID‐19 susceptibility, disease severity, or response to treatment.^[^ ^236^, ^237^ ^]^

However, there are also challenges associated with the use of the N3C platform in precision medicine research. These challenges include the need to protect patient privacy and ensure data security, as well as the need to develop standardized protocols for data collection, analysis, and sharing. Researchers must also carefully consider potential biases in the data and address these biases when analyzing the data.

Second, public health big data can also provide rich medical data, such as genomic data, biomarker data, drug utilization data, etc., supporting the development of personalized medical^[^ ^37^, ^39^ ^]^ and drug treatment plans.^[^ ^238^, ^239^, ^240^ ^]^ For example, using genomic data and drug metabolism enzyme‐related information, healthcare providers can predict a patient's response to certain drugs, select the most suitable treatment plan, and improve treatment effectiveness while reducing adverse reactions.^[^ ^241^, ^242^ ^]^

The outbreak of Coronavirus Disease 2019 (COVID‐19) was caused by the SARS‐CoV‐2 coronavirus, which is highly contagious, spreads rapidly, and accumulates many mutations.^[^ ^243^ ^]^ The emergence of COVID‐19 motivates the need for mathematical modeling methods for rapid understanding of emerging disease outbreaks and finding effective interventions to prevent or mitigate them. Currently, although several studies focus on this direction, most of them model SAS‐CoV‐2 infection at a single scale, either infections at the tissue level^[^ ^244^, ^245^, ^246^ ^]^ or at the population level (i.e., between‐host) to present epidemiological findings.^[^ ^247^, ^248^, ^249^ ^]^ However, many models do not include some important information, such as time‐dependent infectivity and recovery rates, individual age and behavior information, and vaccination. This missing information leads to outcomes of mathematical models that are usually extremely sensitive to input parameters and quite vulnerable and unstable to disturbance.

A multi‐scale system can be designed using multi‐scale mathematical modeling approaches to couple population‐scale dynamics (between‐host) to the events occurring within individual hosts.^[^ ^250^ ^]^ Such a system can be applied for estimating critical epidemiological features of COVID‐19 and its spread in the vaccinated population using all available data such as epidemiological, molecular/genetic, demographic, movement, mRNA vaccination, etc. In the multi‐scale modeling, we can first explore the effect of initial viral load on the severity and duration of COVID‐19 and construct a multi‐component model to shed insight into the dynamics of the disease. By mapping within‐host viral loads onto between‐host transmission parameters, the population data can be modeled across two scales (within‐host and between‐host) to yield a more accurate picture of outbreaks, epidemics, pandemics, and evolution. Two key mathematical models can be used for this purpose. A heterogeneous graph network combined with a susceptible‐exposed‐infectious‐recovered (SEIR) model can be constructed to describe human‐human interactions to measure the impact of people's social behavior on the spread of COVID‐19. Meanwhile, an individual‐based model could be built to help seek appropriate interventions to control COVID‐19. Then, multi‐scale models can be validated, and sensitivity analysis performed to explore which factors have the greatest impact on the onset, duration, and severity of infection. This type of research aims to supply public health officials with tools to understand the epidemiology of COVID‐19 in real time and predict the effect of therapeutic and non‐therapeutic interventions on the course of the disease.

With the emergence of mutated strains such as Delta and Omicron, the cryptic transmission of COVID‐19 cannot be ignored and may bring serious consequences. Currently, a lot of health‐related data needs to be mined from the internet. Better integration and utilization of network big data can improve the sensitivity and timeliness of infectious disease early warning systems, which is an optimization and extension of traditional early warning monitoring research. In the selection of internet big data, previous studies mainly focused on network retrieval data, such as the Google flu index. Nevertheless, it cannot be ignored that people's actual online search behavior is not entirely spontaneous and may be disturbed by other factors, such as media information. In previous studies, some scholars have used the Google search index to analyze the incidence rate of influenza and have detected anomalies. They found that the search index was increased, but no local epidemic occurred. The abnormality was due to the interference effect caused by online media data. Therefore, it is also a popular topic for the application of network retrieval data, population migration data, and network media data to provide early precision warning of the occurrence and development of COVID‐19 cryptic transmission.

Industry‐driven precision medical tools play a vital role in harnessing public health data to deliver precise healthcare services. Such as DeepGestalt, IBM Watson for Genomics, DeepVariant, and so on. These tools integrate bioinformatics, data science, and cutting‐edge technologies, facilitating significant progress in personalized medicine, early disease detection, drug development, public health, and healthcare management. They not only improve treatment outcomes but also reduce healthcare expenses, thereby enhancing patients' overall quality of life. Moreover, these tools propel forward medical science research, expedite drug discovery, and advocate for the adoption of precision medicine, ultimately generating long‐term health advantages for both individuals and society.

In summary, the close relationship between public health big data and precision medicine makes public health data the foundation and support of precision medicine. By fully utilizing public health big data, we can better achieve the goals of precision medicine and provide more personalized and precise medical services for people.

## 10 Security and Privacy of Precision Medicine Data

The Health Information Trust Alliance (HITRUST) is the organization responsible for creating the Common Security Framework (CSF).^[^ ^251^ ^]^ CSF incorporates and cross‐references security requirements from the Health Insurance Portability and Accountability Act (HIPAA),^[^ ^252^ ^]^ Payment Card Industry (PCI),^[^ ^253^ ^]^ National Institute of Standards Technology (NIST),^[^ ^254^ ^]^ Centers for Medicare and Medicaid Services (CMS)^[^ ^255^ ^]^ Information Security Acceptable Risk Safeguards, and CMS Minimum Security Requirements, so we can easily evaluate our compliance with each of these regulations or standards. Using CSF, we have a flexible and scalable “defense in depth” strategy, which allows us to remain current with cyber security technology and standards and address emerging threats. Features of this defense‐in‐depth strategy include 1) security and privacy policy and procedures; 2) new employee and annual training on security and privacy; 3) architecture and infrastructure that implements security at multiple layers from the network perimeter to the desktop; 4) Industry‐leading products such as Tipping Point intrusion prevention systems, Palo Alto next‐generation firewalls and Checkpoint Full Disk Encryption; 5) monitoring of activity and possible incidents using RSA Envision security events and incident monitoring (SEIM) and FairWarning event monitor for HIPAA compliance; 6) risk assessment and vulnerability analysis using the Nessus scanner and HP WebInspect; and 7) commitment to security and privacy demonstrated by hiring a nationally known Chief Information Security Officer (CISO)^[^ ^256^ ^]^ and a formal Privacy and Security Committee with members from Legal, IRB, Compliance, Privacy, IT Security, and HR.

DCA^[^ ^257^ ^]^ can facilitate a centralized, secure, streamlined process for the management of research data requests (and subsequent datasets) made by researchers across individual hospitals. The primary functions of the data control agents are to verify that clinical data requested by researchers from the Investigator Portal is IRB approved and to verify that the datasets generated for the researchers contain only IRB‐approved data elements before they are released. Clinical research datasets generated during this process are stored on secure servers at the medical center. Access is restricted to research staff, Informatics Service Core programmers, and data control agents. Informatics Service Core programmers have access to “working” folders that are not accessible by researchers. Once research datasets are created, they are verified by the data control agents and copied to a secure folder accessible by the researcher.

Privacy protection is one of the primary tasks in precision medical data. Personal identities must undergo anonymization and de‐identification processes to ensure the privacy of patients. Anonymization methods should be adapted to different types of data. Privacy regulations, such as the HIPAA,^[^ ^252^ ^]^ stipulate compliance requirements for patient data. Researchers and healthcare institutions need to adhere to these regulations to prevent unauthorized disclosure of patient information. Maintaining compliance requires regular training and reviews. Additionally, obtaining explicit informed consent before collecting and using patient data is a crucial step in protecting privacy. Patients should be informed about how their data will be used and have the right to refuse or withdraw consent.

The deep learning models based on federated learning play a pivotal role in ensuring the security and privacy of precision medical data.^[^ ^258^, ^259^ ^]^ First, federated learning enables different healthcare institutions or researchers to collaboratively build global models while keeping data local. This avoids centralized storage and transmission of sensitive patient data, thus maximizing privacy protection. Second, federated learning provides a framework for multi‐party data sharing, fostering collaborative research across institutions. Healthcare institutions can jointly train models, resulting in more accurate outcomes, while retaining control over their data. Additionally, federated learning reduces the need for data transfers, lowering the cost of data mobility.

Federated learning will continue to be instrumental in ensuring the security and privacy of precision medical data. In future research, improvements and innovations can be made in the following areas. First, further optimization of federated learning models is possible to handle large‐scale, high‐dimensional biomedical data, ensuring accurate predictions while preserving privacy. Second, promoting the application of federated learning in cross‐border, cross‐domain medical collaborations can enhance our understanding and address global healthcare challenges. Third, integrating different types of medical data, such as images, genomics, and clinical records, into the federated learning framework can provide a more comprehensive perspective.

## 11 Conclusion

With the continuous development and application of big data‐related technologies in the precision medical field, its potential impact on healthcare is enormous. In this paper, we have provided some insights into the technologies and applications of biomedical big data in the field of precision medicine. However, the heterogeneity of data from multiple sources requires us to improve standards for data collection, storage, processing, and sharing. The analysis and utilization of data depend on the progress of key technologies in artificial intelligence. To maximize the value of biomedical big data, it is necessary to properly address challenges from various aspects such as data, algorithms, security, standards, and so on to ensure that it plays a greater role in the field of precision medicine.

## Conflict of Interest

The authors declare no conflict of interest.
